# Childbearing vs. clinical trial participation: is it one or the other?

**DOI:** 10.1186/s12967-021-02930-2

**Published:** 2021-06-15

**Authors:** Sarah J. Hernandez, Lindsay A. Hohsfield

**Affiliations:** 1 Department of Neurobiology and Behavior, University of California, Irvine, Irvine, CA USA; 2 Institute for Memory Impairments and Neurological Disorders, University of California, Irvine, 3400A Biological Sciences III, Irvine, CA 92697-4545 USA

**Keywords:** Autosomal dominant, Neurodegenerative disease, Fertility, Prevention, Clinical trial

## Abstract

Recent advances have shed light on the importance of early therapeutic intervention for neurodegenerative diseases. Primary prevention trials present a potential disease-modifying strategy for pre-symptomatic patients of autosomal dominant neurodegenerative diseases (ADND), such as early onset familial Alzheimer’s disease (AD) and Huntington’s disease (HD). As trials target earlier disease stages, however, prospective participants face new ethical and logistical challenges, namely childbearing and reproductive health decisions. Since pregnancy is an exclusion criteria for such trials, participants of reproductive age must choose between participating in research and having a family. Such decisions carry significant burdens for ADND patients that if left unaddressed could impact patient well-being and the field as whole. We use our perspective as scientists, advocates, and ADND family members to highlight current shortcomings in the field regarding trial participation and family planning issues for ADND patients and call for the establishment of a normative standard to address these concerns.

## Addressing gaps in the ADND field

Extensive study of ADND has led to the discovery of the genes and specific mutations that give rise to these disorders [1, 2]. Despite this, disease-modifying therapies have remained elusive. Subsequent advances and cohort studies have elucidated ADND pathogenesis, which often involves presentation of long pre-symptomatic phases. These observations and the persistent challenges in developing treatments for ADND have led the field to propose primary prevention trials utilizing potential disease-modifying therapies decades before symptom onset. These trials, some of which are underway (clinicaltrials.gov; NCT01760005), will bring new challenges for participants and investigators, namely that participants will be of childbearing age. As primary prevention trials for ADND require foregoing childbearing during participation, participants are faced with difficult, time-sensitive, and complex decisions. These decisions involve navigating the repercussions of joining or not joining a clinical trial, in terms of their future development of disease: the ability to hope a treatment will modify their disease outcome—a cornerstone coping strategy for these disease populations—or holding off on their childbearing plans. ADND researchers must consider the scientific and ethical considerations for including these female subjects as research subjects [3, 4]. It should be noted that these challenges are not limited to ADND patients, and could be implicated in any patient group at high risk for developing a neurological disorder (e.g., autosomal recessive Parkinson’s disease). As young neuroscientists of reproductive age with a family history of early onset familial AD and HD, these challenges are uniquely important to us. However, as ADND patient-associated research has made major contributions to our knowledge of various dementia-related diseases including late onset AD, we believe these challenges are broadly important to the field of neurology, as ADND trials continue to inform our understanding of various neurological disorders.

There are significant gaps in the field regarding the effect of family planning on ADND primary prevention trial participation for both men and women. These include: (1) the paucity of information and study on participant perspectives regarding family planning issues, (2) the lack of guidelines and implementation for family planning communication in trials, (3) the need for ethical consideration regarding informed consent on participant’s fertility-associated physical and emotional well-being, and (4) the need for an assessment of potential risks incurred by trials as well as the ADND and dementia field if these issues go unaddressed. For each of these issues related to childbearing in the ADND population, we highlight the gap within the ADND field, detail findings and strategies employed by the field of oncology to address these issues, and lastly suggest how the approach taken by the field of oncology could serve as a template to guide recommendations for ADND investigators [5]. We acknowledge the existence of apparent differences between the fields of oncology and ADND, including perceived disease risk, decision for treatment, treatment cost benefit, treatment efficacy, and survivorship following treatment. However, we argue that there is commonality between these fields (i.e., undergoing potential disease-modifying treatments that could affect future fertility and patient quality of life) and believe current strategies in oncology could help guide ADND investigators develop patient-centered practices to improve clinical trial participation and care.

## The need for understanding participant perspectives regarding family planning issues

It remains unclear whether experimental treatments for ADND would reduce or eliminate fertility, however, committing to clinical trial participation during critical reproductive years and/or for the remainder of life may cause the loss of optimal childbearing years or push some patients out of their fertility window altogether, impacting patients’ emotional well-being. Due to the lack of data on the effects of potential fertility loss in the ADND community, we have turned to oncology—a field with experience in developing strategies for young patients concerned about their fertility future. Researchers in the cancer field have stressed that understanding the emotional aspects of infertility after cancer as well as the factors that influence a survivor’s decision about having children is of great importance [6]. Cancer survivors who could not preserve their fertility felt increased depression, anxiety, grief, low self-esteem, as well as changes in body image and gender identity [7]. Survivors of inheritable cancer reported more distress about childbearing issues compared to other cancer survivors [6].

**Fig. 1:**
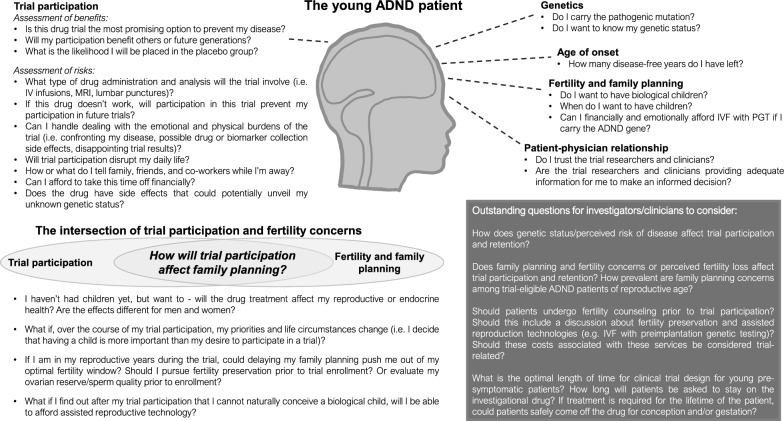
The ADND patient perspective

For the field of ADND, no data exists that captures the attitudes of potential trial participants on family planning issues. To gain a better understanding of patient perspectives as they relate to trial participation and family planning, we suggest that the ADND field undertake such studies. Particularly as the field moves toward treating younger pre-symptomatic and prodromal patients, investigating the experiences and perspectives of ADND patients of reproductive age will identify unmet family planning needs and provide necessary evidence for improved patient-centered care. Perspectives and questions to address (Fig. 1) include: how prevalent are family planning concerns among trial-eligible ADND patients of reproductive age? Would family planning impact trial participation or vice versa? What are the clinical needs and preferences of ADND patients related to fertility counseling and preservation before and throughout trial participation?

## Guidelines: providing an opportunity to discuss fertility-related concerns

No guidelines are currently in place to facilitate discussions with ADND patients about fertility-related concerns regarding their disease or clinical trial participation. Guidelines established by the American Society of Clinical Oncology call for an individualized fertility consultation, including a discussion of fertility preservation options as early as possible and at every stage of treatment for oncology patients that are interested in or uncertain about their future fertility plans prior to cancer treatment [8]. Prior to the establishment of these guidelines, a survey conducted on 904 male cancer patients in a multi-site study reported that 52% of young survivors wanted children, but only 24% banked sperm prior to treatment, citing a lack of provided information as the primary reason [6]. Evidence now shows that providing a specialized fertility counseling session improves patient psychological health and improves quality of life [9]. Even if fertility preservation is not elected, when it is discussed, women report high levels of satisfaction with their fertility-related decision and improved ability to cope with their cancer diagnosis [8].

Given the importance of this issue and the potential impact that loss of fertility can have on quality of life, there is a critical need to provide effective fertility counseling services to ADND patients of reproductive age, particularly before participation in primary prevention trials that require a fertility hold. When establishing future investigational trials, we implore investigators to facilitate conversations between patients, genetic counselors who often have experience with hereditary disorders and associated reproductive questions, and fertility specialists that can help guide family planning decisions (as well as evaluate ovarian reserve/sperm quality prior to trial enrollment). We postulate that taking these measures will improve psychological health, decision satisfaction, and quality of life for trial participants. We endorse the adoption of the recommendations set forth by the American Society of Clinical Oncology to facilitate clinician interactions with ADND patients both as part of trial enrollment and on an ongoing basis during trial participation as patient childbearing concerns evolve and disease-modifying treatments, hopefully, become the standard of care.

## Informed consent: potential risks of trial participation on fertility

There are four basic ethical principles in medicine: autonomy, beneficence, nonmaleficence, and justice. To protect human subjects during clinical research, informed consent is crucial to achieving these principles, which involves informing subjects of their rights, alternative treatment options, risks, and benefits prior to their trial participation. In the ADND field, we believe there is a current gap regarding ethical considerations and patient fertility, particularly informed consent and the measures taken to adequately address and acknowledge the potential risk to trial participants’ fertility during their participation in trials testing investigational treatments with unknown risk. In oncology, studies show that providing information to patients about treatment-associated reproductive risks is integral to helping them make informed decisions [8]. Fertility preservation discussions with health care professionals increases patient understanding of the consequences of their disease/treatment on fertility and elevates patient satisfaction with their health care [7, 9].

In ADND prevention trials, there is limited information on whether investigational drugs undergo testing for fertility-related effects, and whether this data is disclosed to patients. Although these experimental drugs are primarily brain-directed, some prevention trials are currently exploring systemic interventions given orally or intravenously. Thus, it is critically important, and necessary for adequate informed consent, for investigators to provide patients with data on the impact of these experimental drugs on egg supply, sperm health, teratogenicity, and fetal neurodevelopment, or the lack thereof. While an argument could be made that investigational trials should be held until the effects of these drugs on fertility are known, the desire for disease-modifying treatments within the ADND field cannot be overstated. Clinical trial participation forces patients to confront and weigh the importance of many major life situations and decisions: their desire to have biological children, their disease age of onset, their remaining natural fertility window, and the promise of an investigational drug to modify disease pathology (Fig. 1). In addition, if ADND prevention trials include long periods of open label extension or require drug treatment for the lifetime of the patient, patients could lose childbearing capacity due to an extended delay in reproduction (as a result of longer trial participation). This could also result in less disease-free time raising their yet unborn children, reducing quality time spent together. Furthermore, the patient must also consider the chance of their assignment to a placebo group in the investigational trial, and whether contributing to a clinical trial (without receiving potential disease intervention) outweighs the potential loss of fertile years. Without proper discussion of these issues, patient drop-off rates could increase as patients realize their desire to have a biological child outweighs their desire stay in a clinical trial; or patients could feel pressured to stay in a trial longer than they had anticipated and unexpectedly lose out on critical fertility years. Thus, prior to trial enrollment, it is critical to: inform the patient of drug/trial-associated fertility risks, ensure patients understand that treatment-associated fertility holds may overlap with years of successful reproduction, consult patients about fertility preservation options (oocyte/sperm cryopreservation and/or surrogacy)—including a discussion of the costs (both physical and emotional burden) and success rates of assisted reproduction technology (if gamete cryopreservation is chosen), and provide treatment options to facilitate patient pursuance of family planning and childbearing as desired. To ensure that ADND patients have the information necessary to make an informed choice when joining preventative trials, we urge investigators to provide ADND patients with information of temporal and biological trial-related risks to participants’ fertility.

## Risks to the ADND field: potential effects on trial participation

Insight gained from the oncology field may shed light on potential risks that the ADND field may face for investigational treatments. While the effect of fertility concerns on trial participation for the ADND field is currently unknown, 29% of breast cancer patients report that infertility concerns affected their treatment decisions [9], suggesting ADND trials and the field as a whole may face potential risks if fertility-related issues go unaddressed. We recommend a thorough assessment of these potential risks to the field and trial participation. Such risks may include: patient dropout to pursue family planning (due to inadequate informed consent or physical/financial inability to undergo fertility preservation prior to trial enrollment), disruption in the balance of favorable risk-benefit regarding trial participation if reproductive potential is lost, and effect on equal subject selection if certain individuals have an increased ability to preserve their fertility (e.g., wealthy/insured over disadvantaged, male over female).

## Barriers to fertility preservation

Once a patient has decided to pursue fertility preservation, both intrinsic (ethical/moral objection, patient medical literacy, patient-clinician relationship) and extrinsic (provision of resources for fertility preservation, local availability of necessary infrastructure) factors influence the patient’s ability to undergo fertility preservation. Additionally, sex can also be a factor as men and women face different barriers to fertility preservation due to ease/difficulty of sample retrieval and biological ineligibility criteria for women (i.e., age, ovarian reserve, treatment delay required by ovarian stimulation cycle). However, financial burden remains the most significant deterrent against fertility preservation [9], cost for which can vary considerably depending on a patient’s residence and employer healthcare contributions. While genetic testing for ADND is considered a trial-related cost, costs associated with fertility counseling and fertility preservation currently fall on the patients. Thus, ADND investigators could consider building these costs into trial design for future studies. In a study that assessed the impact of fertility cost coverage on female cancer patients, participants described their ability to undergo oocyte cryopreservation as being crucial to their identity, a necessity, and lamented that their inability to do so would have been devastating [10]. No such study exists for ADND patients. While cost will remain a challenge, particularly for disadvantaged or under-insured ADND patients, there are resources for seeking cost coverage for fertility preservation (resolve.org). Until trial design is updated to include fertility-related expenses as a trial-related cost, we encourage investigators to inform patients about these tools, which may prove critical for empowering ADND patients to preserve reproductive capacities and prevent childbearing regret that may result otherwise. Furthermore, fertility preservation options could open the door for patients to pursue assisted reproductive technology that would eliminate the possibility of passing on the disease-causing mutation to their offspring (i.e., in vitro fertilization with preimplantation genetic testing—IVF with PGT).

## Concluding remarks

The distress reported by oncology patients who lost fertility [6] and their anger and resentment from reduced access to fertility preservation [10] may serve as a lesson for the ADND community. Addressing fertility concerns and advocating for fertility preservation in the ADND patient community could signal a commitment to participants of reproductive age regarding their fertility and childbearing concerns and improve patient welfare, ensure autonomy, and maintain respect and trust (see Outstanding Questions in Fig. 1). Given the goal of preventative trials, we encourage investigators to open discussions with patients and trial participants about fertility preservation and the use of IVF with PGT, as many ADND patients remain unaware of this process and the impact it could have both personally for their families and society as a whole. Although out of scope for this article, the possible societal trade-off in economic benefit by eliminating future disease (along with an analysis of the psychological and economic burdens of ADND vs. IVF with PGT within the context of the patient perspective) deserves further discussion. Researchers and ADND families have a history of successful partnership in working together towards the common goal of developing therapies. With the advent of primary prevention trials, we ask that they continue to work together by also considering the implications of trial participation on young ADND patients’ ability to have a family—does it have to be one or the other?

## Data Availability

Not applicable.

## Abbreviations

ADND: Autosomal dominant neurodegenerative disease
AD: Alzheimer’s disease
HD: Huntington’s disease
IVF: In-vitro fertilization
PGD: Preimplantation genetic testing
